# Clinical Presentations and Genetic Characteristics of Late-Onset MADD Due to *ETFDH* Mutations in Five Patients: A Case Series

**DOI:** 10.3389/fneur.2021.747360

**Published:** 2021-11-08

**Authors:** Zhenchu Tang, Shan Gao, Miao He, Qihua Chen, Jia Fang, Yingying Luo, Weiqian Yan, Xiaoliu Shi, Hui Huang, Jianguang Tang

**Affiliations:** ^1^Department of Neurology, Second Xiangya Hospital, Central South University, Changsha, China; ^2^Hunan Key Laboratory of Tumor Models and Individualized Medicine, Second Xiangya Hospital, Central South University, Changsha, China; ^3^Department of Gastroenterology, Second Xiangya Hospital, Central South University, Changsha, China; ^4^Department of Medical Genetics, Second Xiangya Hospital, Central South University, Changsha, China

**Keywords:** *ETFDH*, riboflavin, mutation, late-onset MADD, molecular analysis

## Abstract

**Background:** Late-onset multiple acyl-CoA dehydrogenase deficiency (LO-MADD) describes a curable autosomal recessive genetic disease caused by *ETFDH* mutations that result in defects in ETF-ubiquinone oxidoreductase. Almost all patients are responsive to riboflavin. This study describes the clinical presentations and genetic characteristics of five LO-MADD patients.

**Methods:** From 2018 to 2021, we collected clinical and genetic data on five patients diagnosed with LO-MADD at our hospital and retrospectively analyzed their clinical characteristics, laboratory examination, electromyography, muscle biopsy, genetic analysis, and outcome data.

**Results:** This study included three males and two females with mean onset age of 37.8 years. Fluctuating exercise intolerance was the most common presentation. Serum creatine kinase (CK) levels were significantly elevated in all patients, and plasma acylcarnitine profiles revealed an increase in long-chain acylcarnitine species in three cases. The urinary organic acid study revealed a high level of hydroxyglutaric acid in all patients. Electrophysiology demonstrated myogenic impairment. Muscle biopsies revealed lipid storage myopathy. Molecular analysis identified nine mutations (three novels and six reported) in *ETFDH*. Exercise intolerance and muscle weakness were dramatically improved in all patients treated with riboflavin (100 mg) daily following diagnosis.

**Conclusions:** LO-MADD is caused by *ETFDH* variants and responds well to riboflavin. Three novel *ETFDH* pathogenic variants were identified, expanding their spectrum in the Chinese population and facilitating future interpretation and analysis of *ETFDH* mutations.

## Introduction

MADD, also known as glutaric aciduria type II, is an autosomal recessive inherited metabolic disorder caused by imperfection in either electron transfer flavoprotein (ETF, encoded by *ETFA* and *EFTB*) or ETF-ubiquinone oxidoreductase (ETF-QO, encoded by *ETFDH*). Moreover, the defects lead to abnormalities in electron transportation from acyl-CoA dehydrogenases to the respiratory chain (1–3), hindering the oxidation of fatty acids and amino acids, impairing adenosine triphosphate synthesis, and causing abnormal lipid accumulation in muscle (4–6). The clinical manifestations of MADD are incredibly diverse (7) and are generally classified into three types: neonatal onset with congenital anomalies (type I) or without abnormalities (type II), and late-onset MADD (type III). Type I and II MADD are often fatal with patients frequently dying within a few hours or a few days after birth from complications, such as metabolic decompensation or hypertrophic cardiomyopathy (8).

Most MADD patients are of type III, which tends to have a better prognosis. The initial onset age ranges from 6 months (9) to more than 60 years (10). Furthermore, its severity varies considerably (11–13). Clinical manifestations of LO-MADD patients include muscular symptoms and accompany extramuscular symptoms. In the mainland of China, muscle symptoms are the most common presentations, including muscle weakness and exercise intolerance with or without myalgia (14–16). Regarding extramuscular symptoms, they consist of vomiting, diarrhea, dysphagia, fatty liver, palpitation or shortness of breath, and diastolic cardiac dysfunction (14, 16). A high level of serum creatine kinase (CK) is a non-specific phenomenon of LO-MADD. Elevated short-, medium-, and long-chain acylcarnitine species in blood and altered urinary organic acid levels can also be found in some patients during the acute phase (14). Fortunately, most LO-MADD patients exhibit an excellent therapeutic response to riboflavin, indicating why LO-MADD is also referred to as riboflavin response MADD (RR-MADD) (5, 17).

However, without muscle biopsy and molecular study, these patients are frequently misdiagnosed as having polymyositis and treated with glucocorticoids. Although glucocorticoid therapy could partially improve patient's laboratory indicators or clinical symptoms, its overall efficacy is inferior to riboflavin (18). Here, we describe the clinical and genetic characteristics of five LO-MADD patients.

## Materials and Methods

### Patient Inclusion

We performed *ETFDH* mutation screening in 12 patients with pathologically confirmed lipid storage myopathy (LSM) from June 2018 to January 2021 at the Second Xiangya Hospital. LSM was diagnosed by the appearance of excess lipid droplets in muscle fibers detected by oil red O staining. Additionally, lipid storage caused by glucocorticoid or mitochondrial disease was excluded. Five patients with *ETFDH* mutations were enrolled. All participants provided written informed consent. Additionally, the Ethics Committee of the Second Xiangya Hospital approved this study.

### Biochemical and Muscle Biopsy Studies

Before riboflavin administration, blood, and urine samples from five patients were collected for laboratory examinations, including urinary organic acid and blood acyl-carnitine analyses. After the patients' consent, all patients underwent quadriceps femoris muscle biopsy under local anesthesia. Serial frozen slices of fresh muscle were stained using H&E and Oil Red O (ORO) after being frozen in isopentane and chilled in liquid nitrogen. Additionally, modified Gomori trichrome (MGT), periodic acidic Schiff (PAS), adenosine triphosphatase (ATPase), nicotinamide adenine dinucleotide tetrazolium reductase (NADH-TR), succinate dehydrogenase (SDH), cytochrome c oxidase (COX), and non-specific esterase (NSE) were performed. We fixed these samples sequentially in 2.5% glutaraldehyde and 1% osmium tetroxide and then embedded them in Epon, and finally examined the ultrathin sections using electron microscopy.

### Molecular Studies

We extracted genomic DNA (gDNA) from peripheral blood of subjects using a DNeasy Blood & Tissue Kit (MagPure Buffy Coat DNA Midi KF Kit) according to instructions. Following that, gDNA samples were sent to WUHAN BGI MEDICAL TESTING for the next-generation sequencing (NGS) analysis. We compared the sequences with those in other databases, including NCBI dbSNP, Human Gene Mutation Database (HGMD), 1000 human genome data set, and a database of 100 Chinese healthy adults. The sequences were further analyzed for pathogenicity using several bioinformatics programs, such as CADD, GERP, and MutationTaster. All mutations and potential pathogenic variants were validated using conventional Sanger sequencing methods.

## Results

### Clinical Features

This study included two female and three male participants. All patients had a normal birth and developmental milestones. The average onset age was 37.8 years with a range of 26–50 years, and the average duration between disease onset and confirmation was 9 months. All five patients complained about fluctuating muscle weakness and exercise intolerance, aggravated by a high workload, staying up late, or irregular diet with predominant involvement of proximal extremities (5/5), neck (3/5), throat (3/5), and facial (3/5) muscles. Three patients presented with myalgia. However, no patient suffered from muscle atrophy, encephalopathy, hypoglycemia, or seizure. Ultrasound studies revealed a patent foramen ovale in patient 2 and fatty liver in patient 5. Further details are summarized in Table 1.

**Table 1:** Clinical presentations of five patients with LAMADD in our study.

				**Muscle weakness**												
						**Upper limb**		**Lower limb**								
**No**.	**Gender**	**Onset age (years)**	**Disease duration (month)**	**Neck**	**Masseter**	**Proximal**	**Distal**	**Proximal**	**Distal**	**Myalgia**	**Trigger factor**	**CK (U/L)**	**Mb (μg/l)**	**Electromyography**	**Muscle biopsy**	**Other symptoms**
1	F	40	6	–	–	4	5	4	5	–	Workload/ exercise	387	79.7	MP	LAM	
2	F	50	3	+	+	5	5	4	4	+	Exercise	2,497	1,144	MP	LAM	Patent foramen ovale
3	M	34	12	–	–	5	5	4	5	–	Exercise/cold	1,339	3,24.3	MP	LAM	Fatty liver and ammonia
4	M	40	12	+	+	3	5	3	4	+	Exercise	960	937	MP	LAM	
5	M	25	12	+	+	4	5	3	5	+	Late night/irregular diet	5,990	3,337	MP	LAM	

*F, female; M, male; –, not involved; +, involved; CK, creatine kinase; Mb, myoglobin; MP, myopathic pattern; LAM, lipid accumulation muscle*.

### Biochemical Studies and Muscle Biopsy

Serum CK levels were significantly elevated in all patients, ranging from 2 to 20 times the upper limit of normal (310 μ/l). Meanwhile, lactate dehydrogenase and myoglobin were increased to some extent. At first, two patients were misdiagnosed with polymyositis and treated with glucocorticoids (1.0 mg/kg weight per day). After glucocorticoid treatment, the 50-year-old female (patient 2) had no change in symptoms or laboratory testing. Meanwhile, after steroid treatment, the 26-year-old male (patient 5) with fatty liver and increased liver enzymes exhibited significant decreases in CK, aspartate aminotransferase, lactate dehydrogenase, and blood ammonia. However, the weak muscle strength remained unchanged. For these two patients, glucocorticoid treatment was discontinued once muscle biopsy and molecular analysis revealed MADD. Riboflavin 100 mg per day was administered once following LO-MADD diagnosis.

Urine organic acid analysis revealed excessive levels of 2-hydroxyglutaric acid-3 and 3-hydroxyglutaric-3 in all patients (5/5), whereas some other C5-C10 dicarboxylic acids were increased in three patients (3/5). Blood acylcarnitine analysis revealed that one patient (1/5) had a decreased degree of free carnitine, and three patients (3/5) had a high concentration of long-chain acylcarnitine.

Before riboflavin treatment, electromyography was performed in all patients (5/5), the results revealed a myopathic pattern without nerve conduction deterioration.

**Figure 1:**
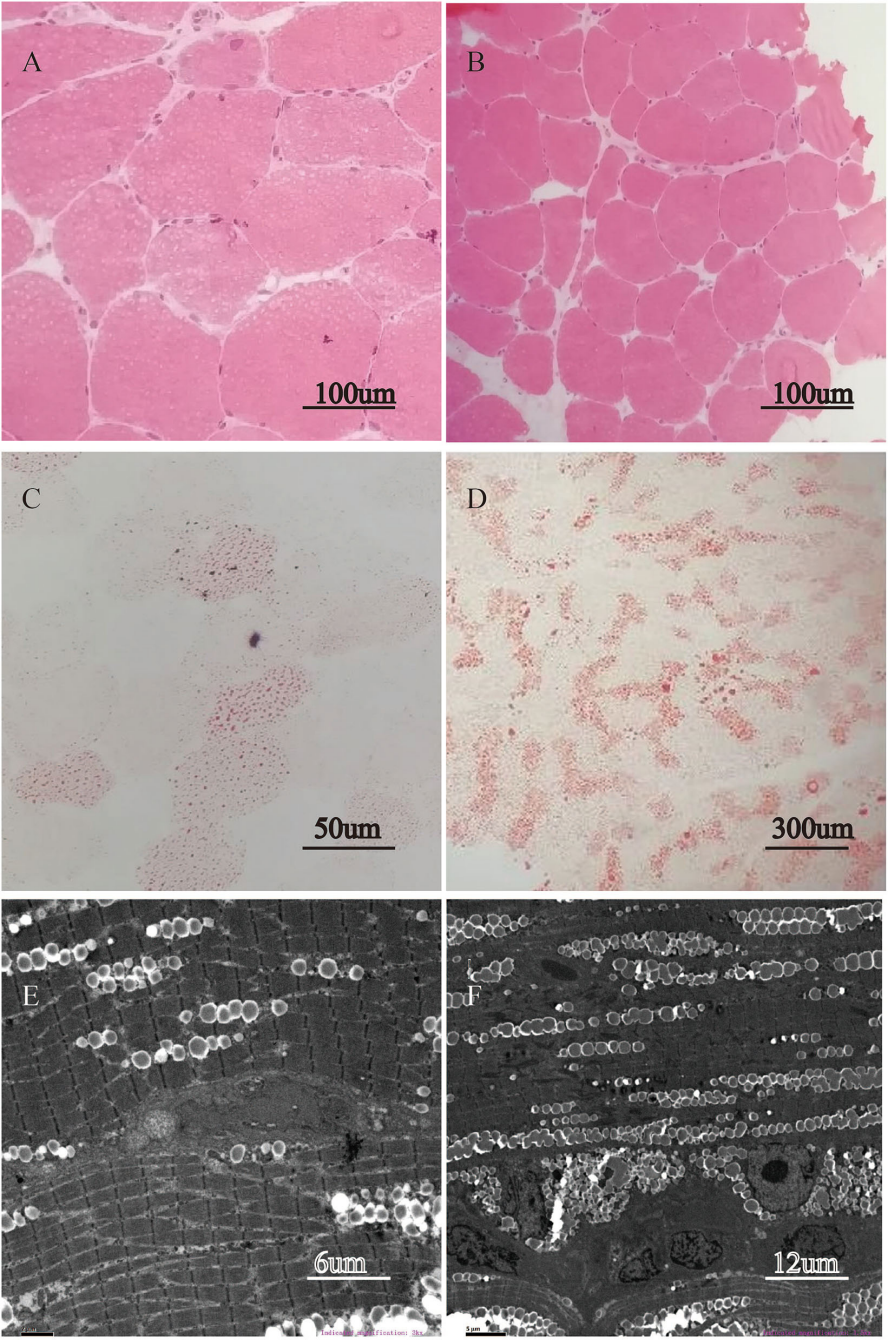
Lipid storage in quadriceps femoris muscle biopsy. H&E stain revealed intracytoplasmic vacuoles appearing in most fibers of patient 2 (**A**, magnification × 400) and patient 3 (**B**, magnification × 200). ORO stain demonstrated abnormal accumulation of lipid droplets in muscle tissue of patient 2 (**C**, magnification × 400), and of patient 3 (**D**, magnification × 60). The electron microscopic ultrastructural muscle fiber study reveals the substantial deposition of lipid droplets between myofibrils in patient 2 (**E**, magnification × 3,000) and in patient 3 (**F**, magnification × 1,500).

Quadriceps femoris muscle biopsy the presence of intracytoplasmic vacuoles on H&E in all five patients as well as a large number of cytoplasmic vacuoles stained with ORO, indicating lipid storage myopathy. In contrast, other stains exhibited no aberration. Under electron microscope, the number of lipids deposited in muscle fibers increased significantly. Here, we used patients 2 and 3 as representative samples in Figure 1.

### Molecular Studies

Nine different *ETFDH* mutations were identified. One patient presented a homozygous mutation (c.770A>G), whereas four exhibited compound heterozygous mutations (c.236C>G and c.1448C>T, c.152G>A and c.524G>T, c.295C>T and c.582_583 ins TA, c.920C>T and c.257G>A) (details in Table 2). The c.152G>A, c.236C>G, c.295C>T, c.524G>T, c.770A>G, and c.1448C>T mutations were reported previously (5, 14, 19–22). Furthermore, c.770A>G was one of the most popular pathogenicity mutations in China (14, 16, 23). Three novel mutations were identified, namely, c.257G>A, c.582-583insTA, and c.920C>T, which are not included in the Human Gene Mutation Database (HGMD) or ClinVar.

**Table 2:** ETFDH gene mutation in five patients in this study.

**No**.	**Exon**	**Location**	**dbSNP**	**HGVSc**	**HGVSp**	**gnomAD**	**Domain**	**GERP_score**	**GERP_pred**	**CADD_score**	**CADD_pred**	**References**
1	3	chr4:159603407	ND	c.236C>G	p.A79G	ND	FAD-binding	5.84	Conserved	25	Damaging	(19)
	10	chr4:159627503	rs377656387	c.1448C>T	p.P483L	0.00002438	4Fe4S cluster	5.03	Conserved	34	Damaging	(5)
2	7	chr4:159616734	rs780015493	c.770A>G Hom	p.Y257C	0.0001	UQ-binding	3.33	Conserved	24	Damaging	(20)
3	2	chr4:159601736	rs534388496	c.152G>A	p.R51Q	ND	FAD-binding	4.79	Conserved	25	Damaging	(14)
	5	chr4:159606289	rs121964955	c.524G>T	p.R175L	0.000004063	FAD-binding	5.73	Conserved	34	Damaging	(21)
4	3	chr4:159603466	rs371493232	c.295C>T	p.R99C	0.00002031	FAD-binding	4.83	Conserved	33	Damaging	(22)
	5	chr4:159606347_159606348	ND	c.582_583insTA	p.P196T fs^*^17	ND	FAD-binding	4.71	Conserved	34	Damaging	Novel
5	8	chr4:159618799	ND	c.920C>T	p.S307F	ND	UQ-binding	5.55	Conserved	33	Damaging	Novel
	3	chr4:159603428	rs777655131	c.257G>A	p.R86H	0.00002031	UQ-binding	3.3	Conserved	24	Damaging	Novel

*Chr, chromosome; ND, no data*.

### Treatment and Follow Up

Since MADD diagnosis was confirmed, riboflavin alone (100 mg daily) was administered, dramatically improving exercise intolerance and muscle weakness within the first week. They later recovered completely following 2–3 months of riboflavin treatment. Furthermore, no further MADD attacks were detected after 2 months to 2 years of follow-up visits.

## Discussion

This study described five LO-MADD patients from the Hunan province of China, exhibiting exercise intolerance and muscle weakness with or without myalgia. The favored sites of involvement include proximal limb, cervical, facial, and throat muscles. Muscle biopsy affirmed abnormal lipid accumulation in myofibers, and electromyography demonstrated myogenic impairment without nerve damage. Anomalies were discovered in blood and urine metabolite tests. Two patients misdiagnosed with polymyositis received glucocorticoids. One of them gained no benefit, and the other showed improvement in laboratory parameters without clinical improvement. Within a few weeks, high-dose riboflavin treatment offered noticeable relief for the five patients.

Consistent with another study (24) and a cohort study published in a Chinese journal about MADD patients from Hunan province, and unlike the coastal areas, two of the three frequent *ETFDH* mutations in China, c.250G>A and c.1227A>C (14, 16, 25) were not prevalent in Hunan. Our result indicates that the mutational spectrum may be heterogeneous in disparate areas. Perhaps the prevalence of c.250G>A mutation is confined to the coastal regions of China, Thailand, and Singapore. As a result, additional epidemiological surveys need to conducted. In addition, three novel mutations in *ETFDH*, including two missense mutations (c.257G>A and c.920C>T) and one frameshift mutation (c.582-583insTA), were identified in merely five cases, indicating that the mutational spectrum of LO-MADD might be broader than that expected in Mainland China.

Similar to previously reported MADD patients, the main clinical manifestations of our patients were exercise intolerance and muscle weakness, which are easily provoked by a high workload, starvation, or infection. ETF-QO protein, a component of the mitochondrial respiratory chain, is responsible for transferring electrons from ETF in the mitochondrial matrix to the ubiquinone pool located in the inner mitochondrial membrane (26, 27), which is critical for fatty acid β-oxidation (FAO). However, RR-MADD–associated mutant ETF-QO, a protein with a gentle structural defect that partly but not wholly disrupts FAD-binding to flavoprotein (1, 2), was unstable and easily degraded by the ubiquitin-proteasome pathway (28). Therefore, the impaired FAO resulted from *ETFDH* mutations impacts energy supply, resulting in lipid accumulation in muscle and induces an increase of its upstream substrates (short-, medium-, and long-chain acylcarnitine) and a decrease in free carnitine, which binds to acylcarnitine. Patients often complain about muscle weakness and exercise intolerance without efficient energy supplements, explaining the male-dominant tendency in some studies (14–16). Due to the remaining function of ETF-QO, RR-MADD patients often present with mild symptoms. By acting as a molecular chaperone to mediate folding defects, the binding of FAD improves the conformational stability of the variant ETF-QO protein (29, 30). As a result, supplementation with riboflavin, the precursor of FAD, upregulates the amount of ETF-QO holoenzyme by producing more FAD that facilitates binding to apoenzymes (31, 32) improving FAO. In addition, glucocorticoids could treat some RR-MADD patients by alleviating oxidative stress associated with MADD (33).

In our study, patient 4 with compound heterozygous variants, a frameshift mutation c.582_583insTA (p.P196T fs^*^17) and a missense variant c.295C>T (p.R99C) had a relatively average age of onset and typical clinical picture of LOMADD. Nevertheless, previous reports reveal that patients with heterozygous frameshift and missense mutations appear to have an earlier age of onset than those with heterozygous missense mutations (32, 34–37). Additionally, they frequently exhibit episodes of vomiting, drowsiness, appetite loss, asthenia, and acetonemic breath (32, 34–37), none of which are prevalent in LOMADD. A high dose of riboflavin (100 mg/day or more) often resulted in excellent improvements for the suffers mentioned previously. In contrast, patients with heterozygous frameshift mutations or homozygosity for null mutations are usually diagnosed with type I or II MADD (38) and are almost unresponsive to riboflavin (8). Interestingly, a 6-month-old girl with a heterozygous frameshift and synonymous mutations, which led to exon 5 skipping and truncated protein production, did not exhibit consistent type I or II MADD symptoms. Instead, the child was diagnosed with LOMADD at the age of 6 months and better responded to riboflavin (9). Therefore, we hypothesize that frameshift mutations are more likely to form truncated proteins, impairing ETF-QO protein function and increasing the likelihood of younger onset age. Nevertheless, the clinical manifestations of frameshift and missense *ETFDH* mutations and their responsiveness to riboflavin remain investigated.

In addition, there is a subset of patients with mutations in riboflavin transporter genes or FAD synthase gene, whose clinical presentation was nearly identical to that of RR-MADD, and dramatic improvement was observed after riboflavin treatment (39). However, genetic analyses revealed no mutation in *ETFDH*, ETFA, or ETFB. In this situation, we should consider mutations of *SLC52A1, SLC52A2, SLC52A3, SLC25A32* (40–43), and *FLAD1* (44) that cause FAD homeostasis disturbance.

Several limitations should be mentioned: (1) We did not measure riboflavin and coenzyme Q10 levels, which might provide some treatment supply indicators, such as whether patients with compound heterozygous frameshift and missense mutations require a higher concentration of riboflavin in blood to recover compared with patients with heterozygous missense mutations. (2) Five cases appear insufficient to present the entire clinical presentation and characteristics of LO-MADD.

In conclusion, the primary clinical presentations of LO-MADD are muscle weakness and exercise intolerance with or without myalgia, and patients better respond to riboflavin. Based on clinical manifestations alone, it is difficult to differentiate the disease from polymyositis and fatty acid oxidation disorders. A final diagnosis requires a combination of blood and urine metabolite analyses, muscle biopsy, and genetic diagnosis. Three novel pathogenic variants of *ETFDH* were identified, enriching their spectrum in the Chinese population and facilitating future genetic data interpretation and analysis.

## Data Availability Statement

The datasets presented in this article are not readily available due to ethical and privacy restrictions. Requests to access the datasets should be directed to the corresponding author.

## Ethics Statement

The studies involving human participants were reviewed and approved by the Ethics Committee of the Second Xiangya Hospital, Central South University. The patients/participants provided their written informed consent to participate in this study. Written informed consent was obtained from the individual(s) for the publication of any potentially identifiable images or data included in this article.

## Author Contributions

ZT conducted the case series and drafted the manuscript. SG, MH, JF, and YL participated in the collection and collation of clinical data. QC and WY conducted the muscle biopsy and pathological analysis. HH and XS were responsible for data analysis of genetic testing and Sanger sequencing. JT and HH were involved in revising the manuscript critically and have given final approval of the version to be published. All authors have read and approved the manuscript.

## Funding

This work was supported by grants Youth Program of the National Natural Science Foundation of China (81901306, MH, 81600996, YL, and 81801123, WY), Huxiang High-Level Talent Gathering Project of Hunan Province (2019RS1011, MH), and Natural Science Foundation of Hunan (2021JJ40857, YL and 2020JJ5810, HH).

## Conflict of Interest

The authors declare that the research was conducted in the absence of any commercial or financial relationships that could be construed as a potential conflict of interest.

## Publisher's Note

All claims expressed in this article are solely those of the authors and do not necessarily represent those of their affiliated organizations, or those of the publisher, the editors and the reviewers. Any product that may be evaluated in this article, or claim that may be made by its manufacturer, is not guaranteed or endorsed by the publisher.
